# Cholangiocyte organoids to study drug-induced injury

**DOI:** 10.1186/s13287-024-03692-6

**Published:** 2024-03-13

**Authors:** Zhenguo Wang, Chen Xing, Luc J. W. van der Laan, Monique M. A. Verstegen, Bart Spee, Rosalinde Masereeuw

**Affiliations:** 1 Division of Pharmacology, Faculty of Sciences, Utrecht Institute for Pharmaceutical Sciences, Utrecht University, Utrecht, The Netherlands; 2 Department of Clinical Sciences, Faculty of Veterinary Medicine, Utrecht University, Utrecht, The Netherlands; 3 Department of Surgery, Erasmus MC Transplant Institute, University Medical Center, Rotterdam, The Netherlands

**Keywords:** Intrahepatic cholangiocyte organoids, Cholangiocytes, Drug induced bile duct injury, Advanced in vitro model, Chlorpromazine, Bile acid, Transporter, Barrier disruption

## Abstract

**Background:**

Drug induced bile duct injury is a frequently observed clinical problem leading to a wide range of pathological features. During the past decades, several agents have been identified with various postulated mechanisms of bile duct damage, however, mostly still poorly understood.

**Methods:**

Here, we investigated the mechanisms of chlorpromazine (CPZ) induced bile duct injury using advanced in vitro cholangiocyte cultures. Intrahepatic cholangiocyte organoids (ICOs) were driven into mature cholangiocyte like cells (CLCs), which were exposed to CPZ under cholestatic or non-cholestatic conditions through the addition of a bile acid cocktail.

**Results:**

CPZ caused loss of monolayer integrity by reducing expression levels of tight junction protein 1 (*TJP1*), E-cadherin 1 (*CDH1*) and lysyl oxidase homolog 2 (*LOXL2*). Loss of zonula occuludens-1 (ZO-1) and E-cadherin was confirmed by immunostaining after exposure to CPZ and rhodamine-123 leakage further confirmed disruption of the cholangiocyte barrier function. Furthermore, oxidative stress seemed to play a major role in the early damage response by CPZ. The drug also decreased expression of three main basolateral bile acid transporters, *ABCC3* (ATP binding cassette subfamily C member 3), *SLC51A/B* (solute carrier family 51 subunit alpha/beta) and multidrug resistance transporter *ABCB1* (ATP binding cassette subfamily B member 1), thereby contributing to bile acid accumulation. CPZ did not induce an inflammatory response by itself, but addition of TNFα revealed a synergistic effect.

**Conclusion:**

These results show that ICOs present a model to identify toxic drugs affecting the bile ducts while providing mechanistic insights into hepatotoxicity.

**Supplementary Information:**

The online version contains supplementary material available at 10.1186/s13287-024-03692-6.

## Introduction

Drug-induced biliary damage is a common feature of drug-induced liver injury (DILI), the leading cause of acute liver failure in the Western world [1, 2]. DILI-associated bile duct damage manifests as cholestatic or mixed type (cholestatic and hepatic) injury, characterized by severe damage to the cholangiocytes causing dysfunction and affecting the architecture of the biliary tree [3]. Cholangiocytes are the epithelial cells that line the intra- and extrahepatic bile duct, and play an essential role in not only the bile composition but also in immune mediation [4], as drug-induced bile duct injury often involves an inflammatory response through the secretion of chemokines and cytokines [5].

Effective and critical assessment of preclinical data could predict potential hepatotoxicity for new chemical entities. Nevertheless, DILI is currently one of the most frequent reasons for drug failure during development and withdrawal from the market. In the past decades, guidelines and predictive in vitro and/or in vivo models that should aid in safety assessment have been reported [6–8]. These models predominantly focus on hepatocyte injury, whereas bile duct injury in the case of DILI is still largely unexplored. It is well known that the majority of drugs and drug metabolites are not only harmful to hepatocytes, but also affect the sinusoidal endothelium and bile duct epithelium [9]. Indeed, several agents have been identified and new mechanisms of bile duct damage have been reported [10, 11]. However, the mechanisms by which drugs induce bile duct injury are diverse and remain poorly understood.

Chlorpromazine (CPZ) is the first-generation anti-psychotic medication on the market, which can cause a clinical manifestation of acute and chronic liver injury [12]. Therefore, hepatotoxicity by CPZ represents a realistic health risk for humans which should not be ignored [13]. Experimental studies revealed that CPZ inhibits bile flow in perfused rat livers [14] and freshly isolated rat hepatocytes [15], a mechanism associated with bile acid accumulation which likely contributes to cholestasis in animals and humans [16]. Furthermore, CPZ-induced hepatotoxicity involves the sustained activation of the c-Jun N-terminal kinases (JNKs) pathway of cellular stress [17], oxidative stress [18], cell-to-cell junction disruption and release of pro-inflammatory cytokines [19]. In this study, we used CPZ to study drug-induced bile duct injury and to mimic cholangiocyte-related cholestasis in vitro.

Previously, we developed a serum-free and chemically-defined condition for intrahepatic cholangiocyte organoid (ICOs) cultures which were differentiated into mature cholangiocytes [20]. These mature cholangiocyte organoids closely resemble the intrahepatic bile duct in vivo, based on the gene and protein expression levels of relevant cholangiocyte markers. Here, we applied the mature cholangiocyte organoid cultures to model CPZ-induced intrahepatic bile duct injury. The aim of this study was to investigate whether the mechanism of CPZ-induced bile duct injury under cholestatic (with the addition of bile acids (BA)) and non-cholestatic conditions can be modelled in vitro using cholangiocyte organoids. Such model could aid in studying DILI and bile duct injury in early phases of drug development and assist in clinical trial design.

## Material and methods

### Organoids and culture conditions

Healthy liver biopsies (n = 5 independent donors, two males and three females) were collected from donor livers during liver transplantation procedures at Erasmus MC-University Medical Center Rotterdam, the Netherlands. The use of the human tissue for research purposes is approved by the Medical Ethical Council of the Erasmus MC-University Medical Center (MEC-2014-060, March 27th, 2018). Anonymous use of redundant human tissue for research purposes is part of the standard treatment agreement with patients in Dutch university hospitals and tissue is used according to the national guidelines ‘code of conduct for the proper secondary use of human tissue’ (www.federa.org). Human intrahepatic cholangiocyte organoid (ICOs) cultures were established from the liver biopsies, as described before [21]. In short, liver biopsies were minced into small pieces and enzymatic dissociation with type II collagenase (0.125 mg/mL; Gibco, Thermo Fisher Scientific, Waltham, MA, USA) and dispase (0.125 mg/mL; Gibco) in DMEM GlutaMAX medium supplemented with DNase I (0.1 mg/mL; Roche, Basel, Switzerland), 1% (v/v) foetal calf serum (FCS; Gibco) and 1% (v/v) penicillin/streptomycin (P/S; Gibco) in a 37 °C water bath. The supernatant was collected, and tissue digestion was performed three times for 10–15 min at 37 °C with fresh enzyme-supplemented medium. The dissociated single cells were then sieved through a 70 µm strainer and rinsed with cold DMEM GlutaMAX medium supplemented with 1% (v/v) FCS and 1% (v/v) P/S and centrifuged for 5 min at 400 g. Subsequently, cell pellets were directly resuspended in cold Matrigel (Corning, New York, NY, USA) and cell-containing droplets were seeded in well plates. After Matrigel gelation, expansion medium (EM) was added, and cells were incubated at 37 °C and 5% (v/v) CO_2_. EM consisted of advanced DMEM/F12 medium (Gibco) supplemented with 1% (v/v) P/S, 1% (v/v) GlutaMax (Gibco), HEPES (10 mM; Gibco), 10% (v/v) Rspondin-1 conditioned medium (the Rspon1-Fc-expressing cell line was a kind gift from Calvin J. Kuo), 2% (v/v) B27 supplement without vitamin A (Invitrogen, Carlsbad, CA, USA), 1% (v/v) N2 supplement (Invitrogen), nicotinamide (10 mM; Sigma-Aldrich, St. Louis, MO, USA), N-acetylcysteine (1.25 mM, NAC; Sigma-Aldrich), fibroblast growth factor 10 (100 ng/mL, FGF10; Peprotech, Rocky Hill, NJ, USA), recombinant human (Leu15)-gastrin I (10 nM, GAS; Tocris Bioscience, Bristol, UK), forskolin (10 µM; Tocris Bioscience), epidermal growth factor (50 ng/mL, EGF; Peprotech), hepatocyte growth factor (25 ng/mL, HGF; Peprotech) and A83-01 (5 µM, transforming growth factor β inhibitor; Tocris Bioscience). Organoids were passaged weekly at 1:3–1:4 ratio and expansion medium was refreshed every 2–3 days.

The differentiation of ICOs to mature cholangiocyte organoids (cholangiocyte-like cell organoids, CLCOs) was performed as described previously [20]. Briefly, ICOs were grown to confluence and mechanically split into small cell clusters and transferred to fresh hydrogel at a ratio of 1:2. The hydrogel consisted of Matrigel mixed with rat-tail type I collagen (1.2 mg/mL; Merck Millipore) at a ratio of 2:3. After allowing the hydrogel to polymerize for 2 h, EM was added. After one day of culture, the EM was changed to defined medium (cholangiocyte differentiation medium, CDM). CDM consisted of Advanced DMEM/F12 medium supplemented with 1% (v/v) P/S, 1% (v/v) GlutaMax, 10 mM HEPES, 2% (v/v) B27 supplement without vitamin A, ITS Premix (5 µg/mL insulin, 5 µg/mL transferrin and 5 µg/mL selenous acid; Corning), 1.25 mM NAC, 100 ng/mL FGF10, 10 nM GAS, 50 ng/mL EGF, 25 ng/mL HGF and 5 µM A83-01. Cells were cultured with CDM for one week and medium was refreshed every other day.

### Preparation of test compounds and bile acid (BA) stock

Chlorpromazine hydrochloride (CPZ; Sigma-Aldrich) solution (0, 10, 20, 30, 40, 50, 80, 160 or 320 μM) was dissolved in the exposure medium (CDM without NAC to reduce antioxidant levels) and prepared freshly before the experiment. The forty- and 80-fold (40×, 80× and 100×) concentrated BA cocktail consisted of five abundant BAs (obtained from Sigma-Aldrich) normally present in human plasma [22–24], shown in Table 1.

**Table 1:** Bile acid concentrations in normal human plasma

Bile acid	Concentration in plasma (μM)	In vitro assay concentration (μM)		
		(40×)	(80×)	(100×)
Glycochenodeoxycholate	1.32	52.8	105.6	132.0
Deoxycholic acid	0.40	15.9	31.9	40.0
Chenodeoxycholic acid	0.39	15.7	31.4	39.0
Glycocholic acid	0.35	14.2	28.3	35.0
Glycodeoxycholic acid	0.31	12.4	24.7	31.0

### Cell viability assay

Cell viability was measured using AlamarBlue™ cell viability reagent (DAL1100; Invitrogen) following the manufacturer’s instructions. Briefly, ICOs were distributed equally in 96 well plates (20 μL hydrogel per well) and differentiated to CLCOs for seven days in CDM. CLCOs were treated with different concentrations of CPZ (0–320 μM) with or without BA cocktail in the exposure medium for 24 and 72 h. After exposure, the stock solution of AlamarBlue cell viability reagent was diluted 1:10 in DMEM/F12 without phenol red (11029-021; Gibco) and sterilized with 0.22 μm filter and prewarmed at 37 °C. The culture medium was removed and replaced with prewarmed sterilized AlamarBlue solution. Organoids were incubated at 37 °C for 90 min, the solution was immediately transferred into new 96 well plates for measurement. The fluorescence intensity (wavelength excitation/emission = 545 nm/590 nm) of the reagents was measured with a microplate reader (CLARIOstar Plus; BMG LabTech).

### Cytotoxicity assay

Cytotoxicity of the compounds were measured using CytoTox 96^®^ Non-Radioactive Cytotoxicity Assay Kit (Promega, Madison, Wisconsin, USA) following the manufacturer’s instructions. The kit is based on lactate dehydrogenase (LDH) which is leaked in the culture medium when the cell membrane integrity is damaged. Briefly, CLCOs were distributed equally in 96 well plates (20 μL hydrogel per well). The CLCOs were treated with different concentrations of CPZ (0–320 μM) with or without BA cocktail (40× and 80×) in the exposure medium for 24 and 72 h. Maximal LDH leakage was measured by lysing CLCOs for 30 min in Cell Lysis Solution. 50 μL of exposure medium was added to 50 μL of substrate reagent and incubated at room temperature for 30 min in the dark. The reaction was terminated by adding 50 μL stop solution and absorbance was measured at wavelength 490 nm with a microplate reader (CLARIOstar Plus; BMG LabTech).

### Intracellular reactive oxygen species (ROS) detection

The generation of intracellular ROS level was measured with 2′,7′-dichlorodihydrofluorescein diacetate (H_2_DCFDA; D399; Invitrogen). H_2_DCFDA was dissolved in ethanol at 10 mM as stock solution and diluted with the DMEM/F12 medium (phenol red free) to 100 μM as working solution. Culture media were removed from the organoids and washed with prewarmed DMEM/F12 medium for 5 min. Organoids were incubated with the H_2_DCFDA working solution at 37 °C for 20 min allowing H_2_DCFDA to enter the cells. After incubation, organoids were washed with the prewarmed DMEM/F12 medium and exposed to compounds at 37 °C for 2 h. The supernatant was removed and CLCOs were washed with prewarmed PBS. Afterwards the organoids were lysed by incubating with lysis solution (90% DMSO and 10% PBS (v/v)) for 1 h on a plate shaker (300 rpm/min). Next, 100 μL lysis solution was transferred to a black opaque 96 well plate to measure fluorescent intensity with a microplate reader (excitation wavelength: 485 nm; emission wavelength: 520 nm).

### Total GSH and GSH/GSSG ratio measurement

The amount of glutathione (GSH) and glutathione disulfide (GSSG) was measured using the GSH/GSSG ratio detection assay kit (ab205811; Abcam; UK) following the manufacturer’s instructions. After the organoids were exposed to the stimulant solution for 24 h, exposure medium was discarded and washed with ice-cold PBS. Organoids were lysed with 400 μL ice-cold 0.5% (v/v) NP-40 in PBS per well and centrifuged at 12,000 g for 15 min at 4 °C. The supernatant was collected and cold trichloroacetic acid was added for deproteinization. The samples were centrifuged at 12,000 g for 5 min at 4 °C. The supernatant was collected and neutralized by adding 1 M NaHCO_3_ solution drop by drop until the pH was 4–6. Then, the samples were centrifuged at 13,000 g for 15 min at 4 °C to remove the trichloroacetic acid and analyzed immediately.

### RNA isolation and RT-qPCR

TRIzol™ Reagent (Invitrogen) was used for extraction of RNA from CLCOs following the manufacturer’s instructions. The concentration and purity of total extracted RNA was measured by the ND-1000 spectrophotometer (NanoDrop, Thermo Fisher Scientific). The synthesis of cDNA was performed using iScript cDNA synthesis kit (Bio-Rad, Hercules, California, USA) according to the manufacturer’s instructions. RT-qPCR was performed to analyze the relative gene expression using the validated primers (Additional file 1: Table S1) following the SYBR Green method (Bio-Rad). The target gene expression levels were normalized to stably expressed reference genes, viz*.* hypoxanthine phosphoribosyltransferase 1 (*HPRT1*), ribosomal protein L19 (*RPL19*) and ribosomal protein S5 (*RPS5*).

### Immunofluorescence analysis

Immunostaining was performed to detect the expression of specific junction proteins. Organoids were fixed with 4% (v/v) paraformaldehyde (Sigma, USA) for 1 h at room temperature. Fixed samples were dehydrated and embedded in paraffin, 5 μm sections were prepared for staining. The deparaffinized and rehydrated sections were incubated with Tris–EDTA (dissolved 1.21 g Tris Base and 0.37 g EDTA in 1L distilled water; pH 9.0) for antigen retrieval at 98 °C 30 min. To avoid non-specific antibody binding, the sections were incubated with 10% (v/v) goat serum in PBS for 1 h and primary antibodies were incubated overnight at 4 °C. After washing with 0.1% (v/v) Tween (Sigma) in PBS and incubated with secondary antibodies for 1 h. Nuclei were stained with DAPI (0.5 μg/mL; Sigma). Finally, sections were washed with PBS three times and fixed with Mounting Medium (Invitrogen). Images were acquired using a Leica (Leica Dmi8; Germany) imaging system. Antibody details are shown in Additional file 1: Table S2.

### ELISA

After compound exposure, cell culture supernatants were collected and stored at − 20 °C. The concentration of inflammatory markers, including IL-6 (88-7066-88, Invitrogen, Carlsbad, CA) and IL-8 (88-8086-88, Invitrogen), were analyzed by ELISA following the manufacturer’s instructions.

### Barrier function assay

To measure the barrier permeability of the CLCOs, a rhodamine 123 (Sigma) leakage assay was performed as previously described [20]. Briefly, organoids were pretreated with 10 μM rhodamine 123 for 2 h at 37 °C, and washed with prewarmed PBS for 5 times. Then organoids were exposed to different concentrations of CPZ and images were captured at different time points (0, 24, 48 and 72 h) with a fluorescence microscope (Leica Dmi8). The luminal fluorescence intensity of organoids was measured and normalized to the background (viz*.* the fluorescence intensity at the outside of organoids) using Image J software (version 1.51j8; National Institutes of Health, USA).

### Statistical analysis

Statistical analysis was performed using GraphPad Prism 9 (GraphPad Software) using one-way ANOVA and paired Student’s t-test. Results were considered statistically significant when *P < 0.05, and details are described in the figure legends.

## Results

### Time and concentration dependent toxicity in organoids exposed to chlorpromazine and bile acids

**Fig. 1:**
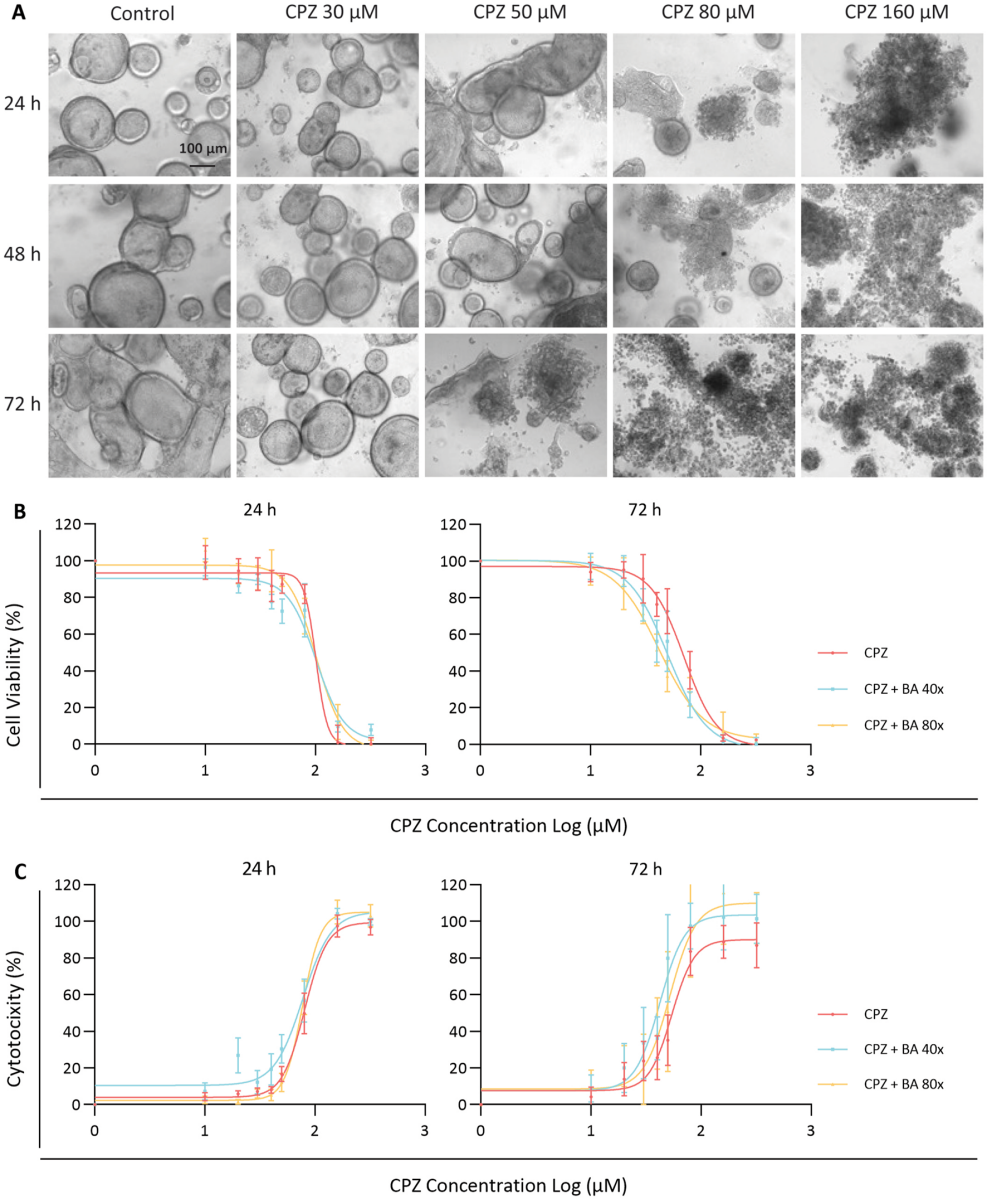
Sensitivity to CPZ and BAs in cholangiocyte-like cell organoids (CLCOs). **A** The morphology of CLCOs incubated with different concentrations of chlorpromazine (0, 30, 50, 80 and 160 μM) for 24, 48 and 72 h. Scale bar = 100 µm. **B**, **C** CLCOs were exposed to a concentration range of CPZ (0, 10, 20, 30, 40, 50, 80, 160 and 320 μM) with non-cholestatic or cholestatic conditions (presence or absence of a BA cocktail (BA 40× or 80×)) for 24 and 72 h. Cell viability was measured by AlamarBlue reagent (**B**), and cytotoxicity was measured by the LDH leakage assay (**C**). Each data point represents the mean ± SEM viability of n = 5 independent donors

We previously demonstrated that liver biopsy (cell pellet) derived cholangiocyte organoids allows the outgrowth of solely LGR5 positive cells as R-spondin promotes the propagation of adult stem cells rather than mature epithelial populations by enhancing canonical Wnt signaling. This was confirmed using cholangiocyte specific markers after establishment of the organoid culture [21] and its differentiation towards bile duct cells [20]. Here, we applied this model in studying cholestasis, a condition during which BAs accumulate in the liver. Cytotoxicity assays were performed by treating the CLCOs with different BA concentrations solely to investigate the cocktail’s toxicity. BA treatment indeed promoted cytotoxicity (*P < 0.05); however, only at the highest concentrations tested (Additional file 1: Fig. S1). Average toxicity induced by the highest concentration BA (100×) was approximately 4.6% after 24 h treatment and near to 5.7% after 72 h treatment. These results are comparable with previous findings demonstrating that cholangiocytes are more resistant to BA toxicity than hepatocytes [25]. To investigate CPZ-induced bile duct injury under cholestatic conditions, organoids were co-incubated with a selected BA cocktail at low (40× BA cocktail) and higher cholestatic conditions (80× BA cocktail). We first evaluated the concentration- and time-dependent cytotoxic effects of CPZ under cholestatic (40× and 80×) or non-cholestatic conditions for 24 and 72 h. After 24 h exposure, CPZ concentrations for up to 30 μM appeared to be non-cytotoxic, as measured by AlamarBlue and LDH release, whereas after 72 h exposure this concentration decreased cell viability and increased LDH release for all conditions tested (Fig. 1B, C). The IC_50_ values of CPZ-induced bile duct injury are presented in Table 2. The effects were accompanied by changes in morphology towards completely disintegrated cholangiocyte-like cell organoids (CLCOs) (Fig. 1A). For subsequent experiments, 30 μM CPZ was used to investigate early and late events leading to the cytotoxic response.

**Table 2:** IC_50_ of stimulation medium in cholangiocyte-like cell organoids (CLCOs)

Compound solution	24 h (μM) (Mean ± SEM)	72 h (μM) (Mean ± SEM)
*Cell viability*		
CPZ	101.1 ± 1.2	70.5 ± 1.1
CPZ + BA 40×	101.2 ± 1.1	50.3 ± 1.1
CPZ + BA 80×	101.7 ± 1.1	42.2 ± 1.1
*Cytotoxicity (LDH release)*		
CPZ	80.6 ± 1.1	54.1 ± 1.1
CPZ + BA 40×	77.8 ± 1.1	42.3 ± 1.1
CPZ + BA 80×	77.2 ± 1.0	52.1 ± 1.1

### Chlorpromazine and cholestasis affect biliary transporter expression levels

**Fig. 2:**
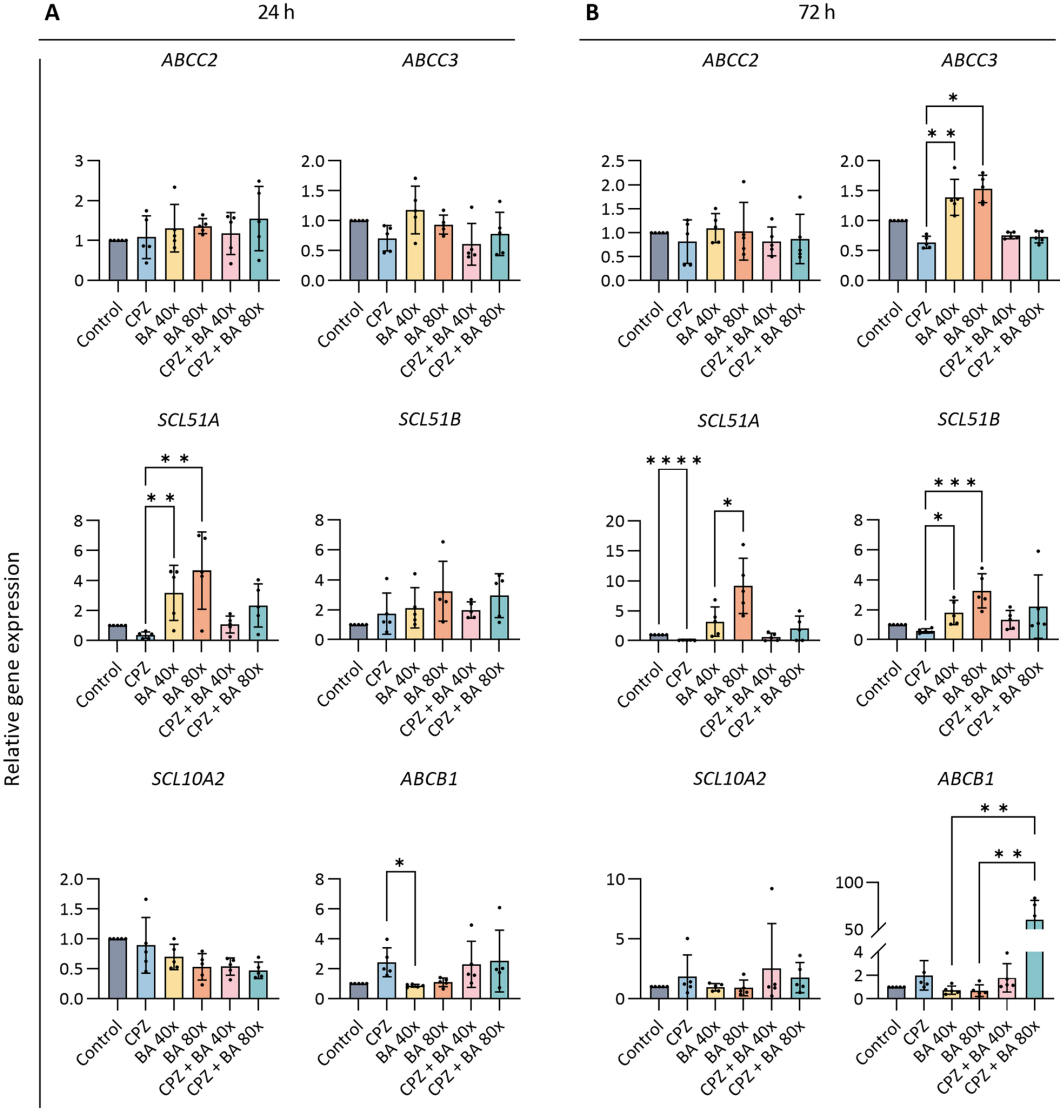
Effects of CPZ and BA cocktail on transporters. **A**, **B** CLCOs were pretreated with 30 μM CPZ with or without BA cocktail (BA 40× or 80×), for 24 (**A**) and 72 (**B**) h. Gene expression of the CLCOs transporters was measured by qPCR and normalized to the housekeeping genes. Data are presented as mean ± SD of five independent donors. Statistical differences between groups were tested using one-way ANOVA followed by Dunn’s test for multiple comparisons; *P < 0.05, **P < 0.01, ***P < 0.001, ****P < 0.0001

Cholangiocytes contain several membrane transporters for BA and drug transport which were evaluated following CPZ exposures under cholestatic and non-cholestatic conditions (Fig. 2A, B). The mRNA levels of the apical transporters ATP-binding cassette sub-family C member 2 (*ABCC2*) and solute carrier family 10 member 2 (*SLC10A2*) were not affected by CPZ exposure under both conditions (cholestatic and non-cholestatic). After 24 h exposure, CPZ exposure slightly down-regulated *SLC51A* and up-regulated *ABCB1* whereas a cholestatic condition upregulated the efflux transporter. In addition, the upregulation of *SLC51A* was counteracted by CPZ co-administration (Fig. 2A). Effects on *SLC51A* and *ABCB1* were more pronounced after 72 h exposure (Fig. 2B). For *ABCC3* and *SLC51B*, the effects were less pronounced after 24 h exposure (Fig. 2A), but after 72 h similar effects as for *SLC51A* were observed (Fig. 2B).

### Chlorpromazine induces oxidative stress in organoids

**Fig. 3:**
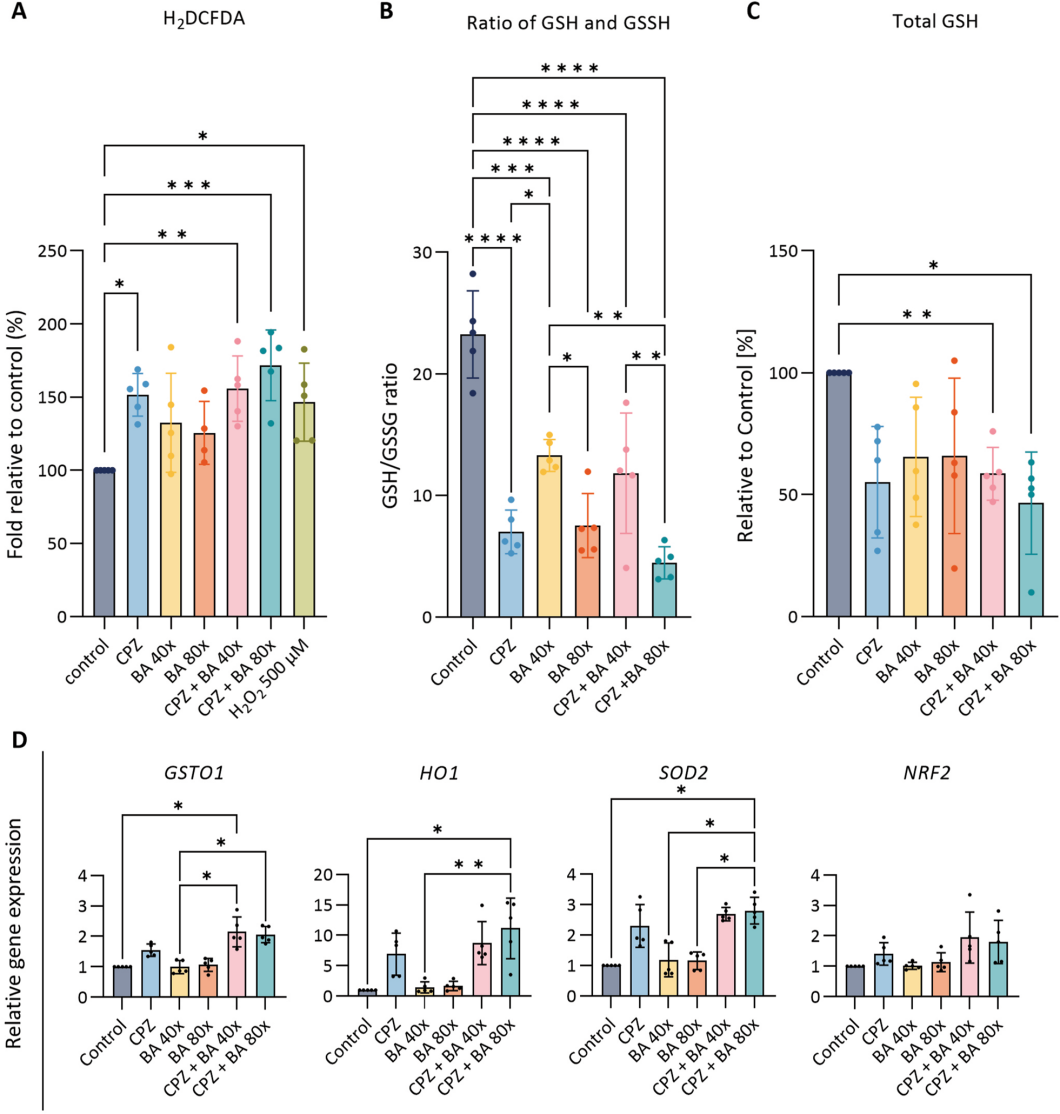
Effects of CPZ and BA cocktail on intracellular generation of reactive oxygen species (ROS) and expression of oxidative stress-related genes. **A** H_2_DCFDA assay in CLCOs. CLCOs were pretreated with 30 μM CPZ with or without BA cocktail (BA 40× or 80×), and 500 μM H_2_O_2_ as positive control for 2 h. Data are presented as mean ± SD of five independent donors. Statistical differences between groups were tested using one-way ANOVA followed by Tukey’s test for multiple comparisons; *P < 0.05, **P < 0.01, ***P < 0.001. **B**, **C** GSH/GSSG (B), total GSH (GSH + 2 GSSG) (C) were measured in CLCOs. CLCOs were pretreated with different conditions (30 μM CPZ with or without BA cocktail (BA 40× or 80×)) for 24 h. Data are presented as mean ± SD of five independent donors. Statistical differences between groups were tested using one-way ANOVA followed by Dunn’s test for multiple comparisons; *P < 0.05, **P < 0.01, ***P < 0.001, ****P < 0.0001. **D** Gene expression of oxidative stress-related genes. CLCOs were pretreated with different conditions (30 μM CPZ with or without BA cocktail (BA 40× or 80×)) for 24 h. Gene expression was measured by qPCR and normalized to the housekeeping genes. Each data point represents the mean ± SD of five independent donors. Statistical differences between groups were tested using one-way ANOVA followed by Dunn’s test for multiple comparisons; *P < 0.05, **P < 0.01

Xenobiotics may generate oxidative stress, which has also been implicated in the early phase and progression of cholestasis [18]. In CLCOs, CPZ-induced ROS generation was investigated under cholestatic and non-cholestatic conditions after 2 h exposure. Compared to the control group, increased ROS production was observed in CPZ treated organoids in absence and presence of the BA cocktail, but the co-incubation did not result in an additive effect (Fig. 3A).

A hallmark of oxidative stress is the depletion of the antioxidant glutathione (GSH), which may exaggerate liver disease in an early stage [26]. Compared to control, the ratio of GSH/GSSG was decreased after CPZ exposure, irrespective of the cholestatic condition, and in the cholestatic conditions alone after 24 h exposure (Fig. 3B). In addition, the total GSH level (GSH + GSSG) was decreased upon exposures with CPZ and BA cocktail suggesting antioxidant depletion (Fig. 3C).

Furthermore, the expression of oxidative stress related genes was analyzed in CLCOs for 24 h exposed to CPZ under cholestatic or non-cholestatic conditions. When compared to untreated controls, CPZ treatment resulted in an upregulation of glutathione S-transferase omega 1 (*GSTO1*), heme oxygenase 1 (*HO1*), transcription factor of NF-E2-related factor (*NRF2*) and superoxide dismutase (*SOD2*) under both cholestatic and non-cholestatic conditions (Fig. 3D).

### Chlorpromazine disrupts barrier function in organoids

**Fig. 4:**
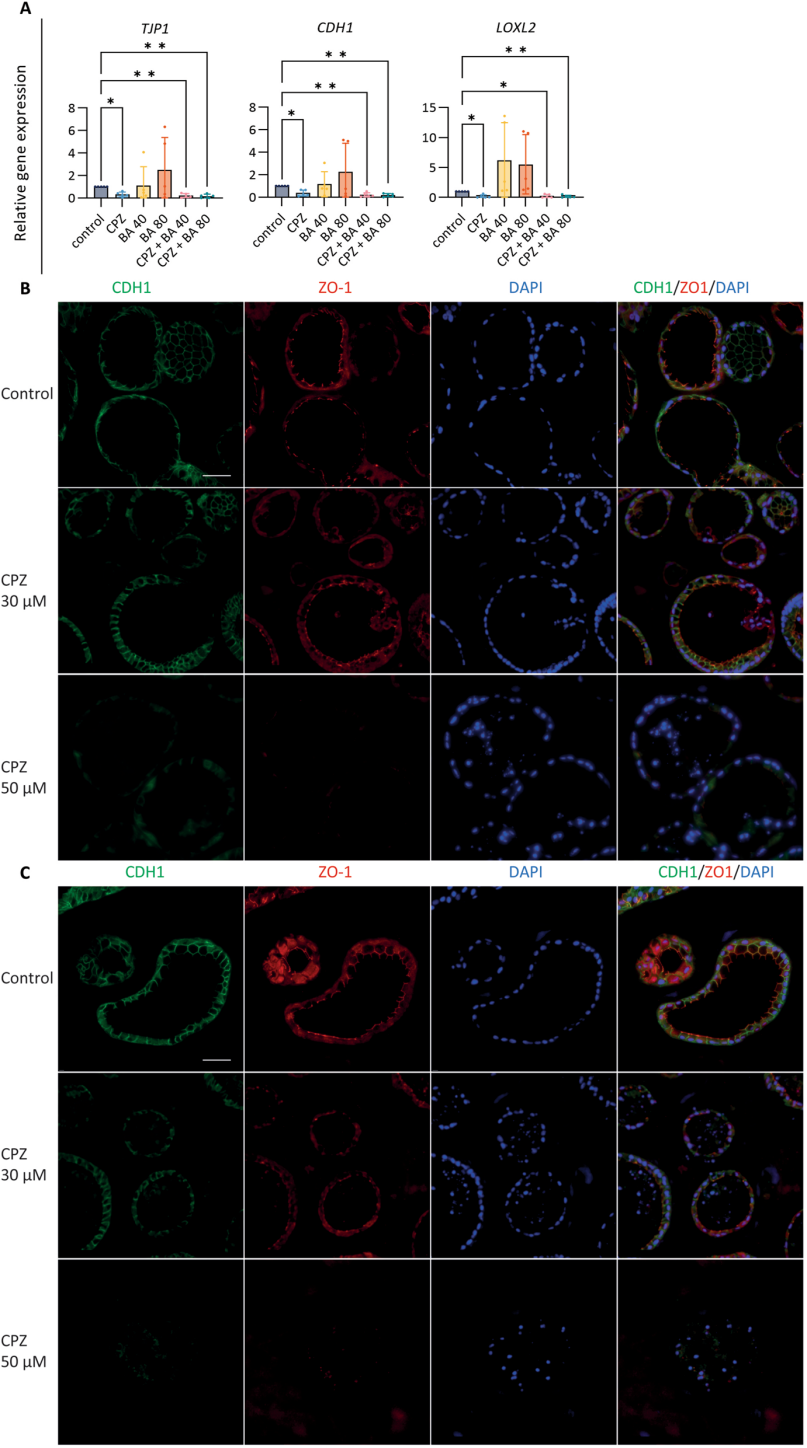
Effects of CPZ and BA cocktail on barrier function. **A** Gene expression of the barrier function-related genes. CLCOs were pretreated with 30 μM CPZ with or without BA cocktail (BA 40× or 80×) for 24 h. Gene expression was measured by qPCR and normalized to the housekeeping genes. Data are presented as mean ± SD of five independent donors. Statistical differences between groups were tested using one-way ANOVA followed by Tukey’s test for multiple comparisons; *P < 0.05, **P < 0.01. **B**, **C** CLCOs barrier disruption assessed by immunofluorescence after CPZ exposure of 24 h (**B**) and 72 h (**C**). Scale bar = 50 µm

Bile duct epithelium plays a crucial role in the formation of a functional barrier to protect hepatic interstitial tissue against the diffusion of toxic substrates from the bile duct lumen. Exposing CLCOs to CPZ led to a reduction in tight junction protein 1 (*TJP1*, also known as ZO1) and E-cadherin 1 (*CDH1*), an effect that was more pronounced under cholestatic conditions (Fig. 4A). Furthermore, lysyl oxidase homolog 2 (*LOXL2*) is an enzyme that belongs to the lysyl oxidase family involved in junction tightness and in extracellular matrix formation by crosslinking collagen and elastin [27]. We observed an upregulation in *LOXL2* in CLCOs after exposure to BA cocktail when compared to control, but a strong down-regulation when exposed to CPZ under cholestatic (40× and 80×) and non-cholestatic conditions (Fig. 4A). Based on these results, we confirmed that CPZ solely and not the BA cocktail disrupted the organoid barrier integrity. Therefore, the next experiments were focused exclusively on CPZ-induced organoid injury.

**Fig. 5:**
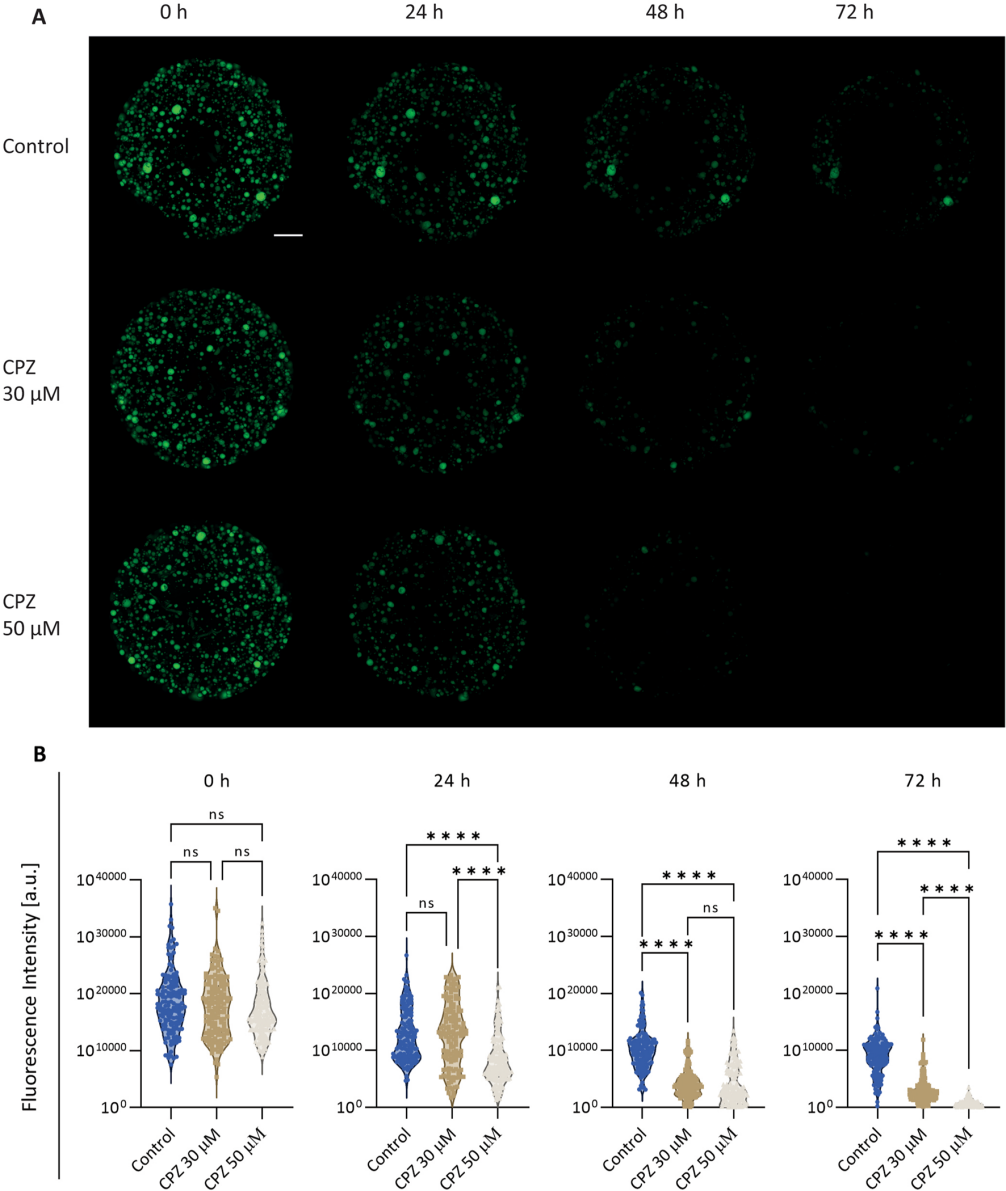
Trans-epithelial barrier function. **A** Fluorescent images show the barrier function by intraluminal fluorescence intensity. Scale bar = 1000 µm. **B** Mean intraluminal fluorescence intensity normalized to background levels for CLCOs. Data are presented as mean ± SD for each group (N = 150 organoids). Statistical differences between groups were tested using one-way ANOVA followed by Dunn’s test for multiple comparisons; ns indicate no significant differences; ****P < 0.0001

To confirm the CPZ-induced effects on barrier function through cell–cell junctions changes, immunostaining of ZO1 and E-Cadherin were evaluated after 24 and 72 h. While CPZ-treated organoids did not show obvious alterations at 30 µM after 24 (Fig. 4B) and 72 h (Fig. 4C), a higher concentration of 50 µM led to cell–cell junction degradations in a time-dependent manner (Fig. 4B, C). For functional assessments, the efflux of rhodamine 123 from the organoid’s lumen was evaluated. In healthy organoids, rhodamine 123 is taken up basolaterally from the medium and then excreted apically by MDR1, resulting in the accumulation of fluorescent dye in the organoid lumen, as previously described [20]. We observed that the fluorescence intensity decreased in the control group over time, likely due to quenching by the laser light exposure. However, CPZ treatment showed a significant loss of rhodamine from the organoid’s lumen in a concentration- and time-dependent manner when compared to control. These results suggest that CPZ affects the cholangiocyte barrier function (Fig. 5A, B).

### Chlorpromazine and TNFα in proinflammatory response

The innate immune response plays an important role in DILI. To evaluate this, we studied CPZ induced inflammation under cholestatic and non-cholestatic conditions. To this end, organoids were treated with CPZ or CPZ and the BA cocktail for 24 and 72 h, after which pro-inflammatory and anti-inflammatory cytokines were measured. The chemokine ligands 4 (*CCL4*) displayed a downregulation under CPZ-treated cholestatic and non-cholestatic conditions when compared to control. Other inflammatory markers were mostly unaffected on the mRNA level (Additional file 1: Fig. S2A, B). Furthermore, IL-6 and IL-8 were not released (Additional file 1: Fig. S3A, B).

**Fig. 6:**
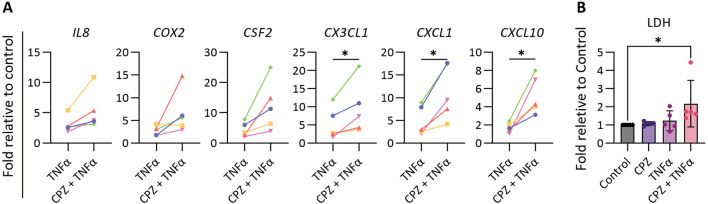
Effects of CPZ and TNFα in proinflammatory response. **A** Gene expression of the proinflammatory cytokine-related genes. CLCOs were pretreated with 30 μM CPZ and/or 30 ng/mL TNFα for 24 h. Gene expression was measured by qPCR and normalized to the housekeeping genes. Data are presented as mean ± SD of five independent donors. Statistical differences between groups were tested using paired t test; *P < 0.05. **B** Cytotoxicity of CPZ-TNFα. CLCOs were pretreated with 30 μM CPZ and/or 30 ng/mL TNFα for 24 h. Cytotoxicity was measured by the LDH assay. Data are presented as mean ± SD of five independent donors. Statistical differences between groups were using one-way ANOVA followed by Dunn’s test for multiple comparisons; *P < 0.05

We next investigated the synergistic effect of the cytokine TNFα on the CPZ induced effects in CLCOs after 24 h exposures. The proinflammatory prostaglandin-endoperoxide synthase 2 (*PGTS2*), colony stimulating factor 2 (*CSF2*), chemokine (C-X3-C motif) ligand 1 (*CX3CL1*), chemokine (C-X-C motif) ligand 1 (*CXCL1*) and C-X-C motif chemokine ligand 10 (*CXCL10*) were all upregulated under the combined TNFα-CPZ treatment when compared to TNFα only treated group (Fig. 6A).

To confirm that inflammation enhances CPZ-induced bile duct injury, organoids were treated with CPZ, TNFα or combined for 24 h, and cytotoxicity was evaluated through LDH leakage. LDH was increased in the combined treatment compared to CPZ or TNFα alone (Fig. 6B). This suggests that inflammation could enhance drug induced biliary injury.

## Discussion

DILI is a risk factor in the clinics and an important cause of withdrawal during drug development. Drug-induced bile duct injury is usually resolved by a few days up to a few months following drug discontinuation, but the time to recovery is often not known and could be indefinite. Therefore, bile duct injury can prolong and even persist, which may lead to tissue degeneration and loss of the bile duct [28]. Although rare, the clinical manifestation of drug-induced bile duct injury is often complex and unpredictable, with secondary biliary cirrhosis, liver failure and eventually the need for transplantation as an outcome [10]. The organoid model developed in the current study could potentially bridge the knowledge gap in revealing underlying mechanisms of drug-induced bile duct injury and may be useful for applications in preclinical drug development. ICOs have been recently described as a useful model in applications such as tissue engineering [29], evaluation of liver function [30] and toxicity testing [31]. Here, we investigated the potential utility of ICO-derived cholangiocyte-like cell organoids (CLCOs) in drug-induced bile duct injury by testing for toxicity, functional transport, ROS production, barrier integrity and inflammatory response.

In vitro modelling of the human bile duct has proven useful for functional (*e.g.* BA secretion) and toxicological studies. For this, the biliary epithelial cell lines, H69, MMNK-1 and HepaRG presented key biliary markers and have been used to mimic the cholangiocyte phenotype [32]. Furthermore, primary cholangiocytes possess the morphological and functional characteristics of the original tissue, but the primary sources are scarce and rapidly dedifferentiate in (two-dimensional) culture conditions, resulting in reduced FXR expression [33] that indicate the loss of specific functions including BA homeostasis [34]. Tumor-derived cell lines play an important role in studying cholangiocarcinoma biology and anticancer therapeutics [35]; however, without a defined function [36] they are not suitable to study drug toxicity. For this, the human iPSCs-derived cholangiocyte-like cells were used, but studies were hampered by time consuming and low efficiency processes [37]. Cholangiocyte organoids retain most characteristics and the physiological structure of native cholangiocytes, and therefore offer a promising strategy for drug innovation [20].

Cholangiocytes contribute to BA reabsorption and bile secretion via the concerted action of membrane transporters that support the enterohepatic circulation of bile salts. Consequently, a holistic in vitro model should include the variety of BAs that could accurately predict the drug-induced bile duct injury under cholestatic and non-cholestatic conditions. Due to the limited information available on bile acids composition present in bile duct, we selected a cocktail of five major BAs present in plasma [22–24], which was described earlier to study cholestasis in hepatocytes [38, 39]. Previously, a 60-fold concentrated BAs mixture did not affect urea formation in a hepatocyte-based cell while it appeared suitable to model drug-induced cholestasis [38]. Therefore, 40-fold and 80-fold concentrated BA cocktail conditions were selected to model mild and severe cholestasis in our research. To further model drug-induced cholestasis, CPZ was selected which has been reported to be associated with such type of liver injury in vivo [13] and in vitro using hepatic 3D spheroids and prolonged exposures (8 to 14 days) [39]. Consistent with our findings, a short exposure (24 h) of CLCOs to CPZ in the presence of concentrated BA cocktail did not reveal a synergistic toxicity. However, extending the exposure to 72 h resulted in a mildly increased effect of the BAs cocktail on CPZ-induced toxicity. Cholestatic DILI has been associated with BA homeostasis in hepatocytes, which is tightly regulated by membrane transporters and metabolic enzymes. Therefore, any drug-related effect on these transporter and enzyme systems can potentially lead to the accumulation of BAs and/or xenobiotics, which may induce liver injury.

In hepatocytes, BAs are taken up by sodium taurocholate co-transporting polypeptide (NTCP, *SLC10A1*) and by organic anion transporting polypeptides (OATP, *SLCO*) from the portal blood. The excretion of BAs into bile canaliculi is mediated by the bile salt export pump (BSEP; *ABCB11*) and multidrug resistance associated proteins (MRP2, *ABCC2*; MDR3, *ABCB4*). BSEP inhibition has been implicated in liver failure [40], and used in in vitro models to predict drug-induced cholestasis [41]. CPZ-induced BA accumulation appeared to inhibit *ABCB11* expression [39, 42]. However, the structure and function of cholangiocytes are different from hepatocytes, as BAs are actively taken up by the apical sodium-bile acid transporter (ASBT; *SLC10A2*) and the basolateral truncated ASBT (t-ASBT), multidrug resistance protein 3 (MRP3, *ABCC3*) and organic solute transporters OSTα/β (*SLC51A/B*) return to the hepatocytes via the cholehepatic shunt [43]. We hypothesized that one of the toxic mechanisms of CPZ is a disruption of cholangiocytes BA homeostasis. Analysis of the transporter’s expression in CLCOs revealed that *SLC51A/B* and *ABCC3* were down-regulated by CPZ treatment, which might contribute to CPZ induced cholestasis in cholangiocytes. Interestingly, we observed that the mRNA levels of these transporters were upregulated by BAs in absence of CPZ. In agreement to a previous report, BAs seem to play a critical role in both the initiation and recovery processes of DILI [44].

The toxicity of CPZ could be mediated by oxidative stress [18, 39] but the mechanism underlying the action of CPZ, alone or in combination with BAs, in bile ducts was not known. Our study confirms that CPZ-induced bile duct injury is associated with increased oxidative stress. Furthermore, GSH plays an essential role in maintaining redox homeostasis and protecting cells from ROS. The decreased GSH/GSSG ratio by CPZ with or without BAs underlines the apparent oxidative stress, which was further confirmed by an upregulation of related genes, viz*. NRF2* exerts antioxidant activity by eliminating ROS and its target genes *HO1*, *GSTO1* and *SOD2* play a role against oxidative stress.

CPZ as anti-psychotic medication, reaches a plasma concentration of approximately 271 µg/L (0.85 µM) in patients [45], which has been reported to induce bile duct injury and to show clinical symptoms of inflammation [13]. In this study, we used a higher concentration, as the intrahepatic concentration of CPZ has not been reported for humans. More importantly, these higher concentrations of CPZ used are in line with those used in other studies [18, 19]. Furthermore, a higher concentration (50 µM) of CPZ induced an upregulation in TNFα and IL6 [19]. Drug-induced bile duct injury is often associated with an immune response [18, 45]. In our study, multiple genes involved in inflammatory responses were not affected by CPZ, individually or combined with BAs, but this is in line with studies in HepaRG cells using a low drug concentration (20 µM CPZ) [46]. Previous evidence suggested that xenobiotic exposure during inflammation can increase an individual’s susceptibility to toxicity [47]. Interestingly, combining CPZ with TNFα generated an injury response and the release of inflammatory cytokines as cholestatic features [46, 47]. Moreover, CPZ-induced oxidative stress was associated with an impairment of F-actin cytoskeleton and tight-junction protein disruption in a hepatocyte-based in vitro model [18, 19, 39]. Inhibition of LOXL2 follows the onset of liver fibrosis and augments collagen degradation [48], which influences tissue stiffness and resilience [49]. In addition, knockdown of LOXL2 induced apoptosis and cell cycle arrest in liver cancer stem cells [50]. These results indicated that CPZ may lead to barrier disruption of the cholangiocytes, regardless of the cholestatic or non-cholestatic condition. Assessment of the barrier function in addition to immunostaining confirmed the presence of these toxic events in our organoid model.

## Conclusion

In conclusion, the present work provides the first in vitro model of bile duct injury using CLCOs. CPZ-induced dose- and time-dependent damage to CLCOs under both normal and cholestatic conditions. CPZ alone did not induce an inflammatory response, but combined with TNFα a clear effect was observed. Oxidative stress caused by CPZ together with the inhibition of efflux transporter (*SLC51A*/*B* and *ABCC3*) expression indicate that CPZ might inhibit bile acid efflux to induce cholestatic features. In the future, this model might be useful in preclinical safety testing to predict drug-induced bile duct injury.

### Supplementary Information

**Additional file 1: Table S1.** List of primers for gene expression analyses. **Table S2.** List of antibodies for immunofluorescence. **Figure S1.** Cytotoxicity of BA cocktail in cholangiocyte-like cell organoids (CLCOs). LDH release as cytotoxicity read-out after exposure of CLCOs to various concentrations of the BA cocktail (for composition see Table 1 of the main manuscript) for 24 and 72 h. Data are presented as mean ± SD of five independent donors. Statistical differences between groups were determined using one-way ANOVA followed by Tukey’s test for multiple comparisons; *P < 0.05. **Figure S2.** Effects of CPZ and BA cocktail on the proinflammatory gene expression. **A**, **B** CLCOs were pretreated with solvent (control), 30 μM CPZ with or without BA cocktail (BA 40× or 80×) for 24 (**A**) and 72 h (**B**). Gene expressions were estimated by qPCR and normalized to the housekeeping genes. Data are presented as mean ± SD of five independent donors. Statistical differences between groups were using one-way ANOVA followed by Tukey’s test for multiple comparisons; *P < 0.05. **Figure S3.** Effects of CPZ and BA cocktail on the proinflammatory release. **A**, **B** CLCOs were incubated with 30 μM CPZ with or without BA cocktail (BA 40× or 80×) and medium were estimated by ELISA, IL6 and IL8 were tested by 24 (**A**) and 72 h (**B**). Data are presented as mean ± SD of five independent donors. Statistical differences between groups were using one-way ANOVA followed by Tukey’s test for multiple comparisons.

## Data Availability

The datasets used and/or analyzed during the current study available from the corresponding author on reasonable request.

## Abbreviations

ABCB1: ATP binding cassette subfamily B member 1
ABCC2: ATP-binding cassette sub-family C member 2
ABCC3: ATP binding cassette subfamily C member 3
ASBT: Apical sodium-bile acid transporter
A83-01: Transforming growth factor β inhibitor
BA: Bile acids
BSEP: Bile salt export pump
CCL4: Chemokine ligands 4
CDH1: E-cadherin 1
CDM: Cholangiocyte differentiation medium
CLCs: Cholangiocyte like cells
CLCOs: Cholangiocyte-like cell organoids
CPZ: Chlorpromazine
CSF2: Colony stimulating factor 2
CX3CL1: Chemokine (C-X3-C motif) ligand 1
CXCL1: Chemokine (C-X-C motif) ligand 1
CXCL10: C-X-C motif chemokine ligand 10
DILI: Drug-induced liver injury
EGF: Epidermal growth factor
FCS: Foetal calf serum
FGF10: Fibroblast growth factor 10
GAS: Recombinant human (Leu15)-gastrin I
GSH: Glutathione
GSSG: Glutathione disulfide
GSTO1: Glutathione S-transferase omega 1
H_2_DCFDA: 2′,7′-Dichlorodihydrofluorescein diacetate
HGF: Hepatocyte growth factor
HO1: Heme oxygenase 1
HPRT1: Hypoxanthine phosphoribosyltransferase 1
ICOs: Intrahepatic cholangiocyte organoids
IL-6: Interleukin 6
IL-8: Interleukin 8
ITS Premix: Insulin, transferrin and selenous acid
JNKs: C-Jun N-terminal kinases
LDH: Lactate dehydrogenase
LOXL2: Lysyl oxidase homolog 2
MRP: Multidrug resistance associated proteins
NAC: *N*-Acetylcysteine
NRF2: Transcription factor of NF-E2-related factor
NTCP: Sodium taurocholate co-transporting polypeptide
OATP: Organic anion transporting polypeptides
P/S: Penicillin/streptomycin
PGTS2: Prostaglandin-endoperoxide synthase 2
ROS: Reactive oxygen species
RPL19: Ribosomal protein L19
RPS5: Ribosomal protein S5
SLC10A2: Solute carrier family 10 member 2
SLC51A/B: Solute carrier family 51 subunit alpha/beta
SOD2: Superoxide dismutase
TJP1: Tight junction protein 1
TNFα: Tumor necrosis factor α
