# Supplementary material for: Cholangiocyte organoids to study drug-induced injury
Source: Stem Cell Res Ther. 2024 Mar 13;15:78. doi: 10.1186/s13287-024-03692-6 (PMC10935784; doi:10.1186/s13287-024-03692-6)
Supplement: Supplementary file 1 — Additional file 1: Table S1. List of primers for gene expression analyses. Table S2. List of antibodies for immunofluorescence. Figure S1. Cytotoxicity of BA cocktail in cholangiocyte-like cell organoids (CLCOs). LDH release as cytotoxicity read-out after exposure of CLCOs to various concentrations of the BA cocktail (for composition see Table 1 of the main manuscript) for 24 and 72 h. Data are presented as mean ± SD of five independent donors. Statistical differences between groups were determined using one-way ANOVA followed by Tukey’s test for multiple comparisons; *P < 0.05. Figure S2. Effects of CPZ and BA cocktail on the proinflammatory gene expression. A, B CLCOs were pretreated with solvent (control), 30 μM CPZ with or without BA cocktail (BA 40× or 80×) for 24 (A) and 72 h (B). Gene expressions were estimated by qPCR and normalized to the housekeeping genes. Data are presented as mean ± SD of five independent donors. Statistical differences between groups were using one-way ANOVA followed by Tukey’s test for multiple comparisons; *P < 0.05. Figure S3. Effects of CPZ and BA cocktail on the proinflammatory release. A, B CLCOs were incubated with 30 μM CPZ with or without BA cocktail (BA 40× or 80×) and medium were estimated by ELISA, IL6 and IL8 were tested by 24 (A) and 72 h (B). Data are presented as mean ± SD of five independent donors. Statistical differences between groups were using one-way ANOVA followed by Tukey’s test for multiple comparisons. [file 13287_2024_3692_MOESM1_ESM.docx]

Supplementary Material

# Supplementary Tables

| **Supplementary Table S1. List of primers for gene expression analyses** | | |
| --- | --- | --- |
| **Gene** | **Forward primer** | **Reverse primer** |
| *RPL19* | ATGAGTATGCTCAGGCTTCAG | GATCAGCCCATCTTTGATGAG |
| *HPRT1* | TATTGTAATGACCAGTCAACAG | GGTCCTTTTCACCAGCAAG |
| *RPS5* | TGCAGGATTACATTGCAGTG | CATCATGGAGTTAGTGAGGC |
| *ABCC2* | GCCAACTTGTGGCTGTGATAGG | ATCCAGGACTGCTGTGGGACAT |
| *ABCC3* | GTCCGCAGAATGGACTTGAT | TCACCACTTGGGGATCATTT |
| *ABCB1* | AATGATGCTGCTCAAGTTAAAGGG | TCAGTAGCGATCTTCCCAGAACC |
| *SLC51A* | TTGTTCGCCTCCCTATTCC | TTGTGGTCTTTCCTTCGGT |
| *SLC51B* | TGTGGTGGTCATTATAAGCATGG | TCTTAGGTTGTTTAGGCTGTTGTG |
| *SLC10A2* | CTGTGCCTCCTTATCTATACCA | AGAGAAACCAGAGATGTACCT |
| *GSTO1* | AGGACGCGTCTAGTCCTGAA | TTCCCTGGGTATGCTTCATC |
| *HO1* | ACTTTCAGAAGGGCCAGGT | TTGTTGCGCTCAATCTCCT |
| *SOD2* | CGTTGGCCAAGGGAGATGTT | CAGCAACTCCCCTTTGGGT |
| *NRF2* | TCAGCATGCTACGTGATGAAG | TTTGCTGCAGGGAGTATTCA |
| *TJP1* | AAGTCACACTGGTGAAATCC | CTCTTGCTGCCAAACTATCT |
| *CDH1* | AGGCCAAGCAGCAGTACATT | ATTCACATCCAGCACATCCA |
| *LOXL2* | CAAGCCAGAGCAACCCCTGGT | CTCGTTGAGGTGGATGGGTC |
| *IL 1β* | TTCGAGGCACAAGGCACAA | TGGCTGCTTCAGACACTTGAG |
| *TNFα* | GCCGCATCGCCGTCTCCTAC | AGCGCTGAGTCGGTCACCCT |
| *COX2* | GGGAACACAACAGAGTATGC | TCTCCTATCAGTATTAGCCTGC |
| *CX3CL1* | GCAAACGCGCAATCATCTTG | ATCTGCTTCTCGAAGGTGCC |
| *IL8* | CAAGAGCCAGGAAGAAACCA | TCTAAGTTCTTTAGCACTCCTTGG |
| *CXCL2* | AGCTTGTCTCAACCCCGCA | GTCAGTTGGATTTGCCATTTTTCAG |
| *CCL4* | CCCAGCCAGCTGTGGTATTC | CCTGGACCCAGGATTCACT |
| *CSF2* | GAGACACTGCTGCTGAGATGA | CAGGAAGTTTCCGGGGTTGG |
| *CXCL1* | AGCTCTTCCGCTCCTCTCA | CACGGACGCTCCTGCTG |
| *CXCL10* | CCACGTGTTGAGATCATTGCT | TGCATCGATTTTGCTCCCCT |

| **Supplementary Table S2. List of antibodies for immunofluorescence** | | | | |
| --- | --- | --- | --- | --- |
| **Antibody** | **Species** | **Supplier** | **Cat. No** | **Dilution** |
| ZO1 | Rabbit | Invitrogen | 40-2300 | 1:250 |
| E-cadherin | Mouse | BD Bioscience | 610181 | 1:100 |
| Anti-mouse Alexa 488 | Goat | Thermo Fisher | A-11029 | 1:200 |
| Anti-Rabbit Alexa 568 | Goat | Thermo Fisher | A-11036 | 1:200 |

# Supplementary Figures


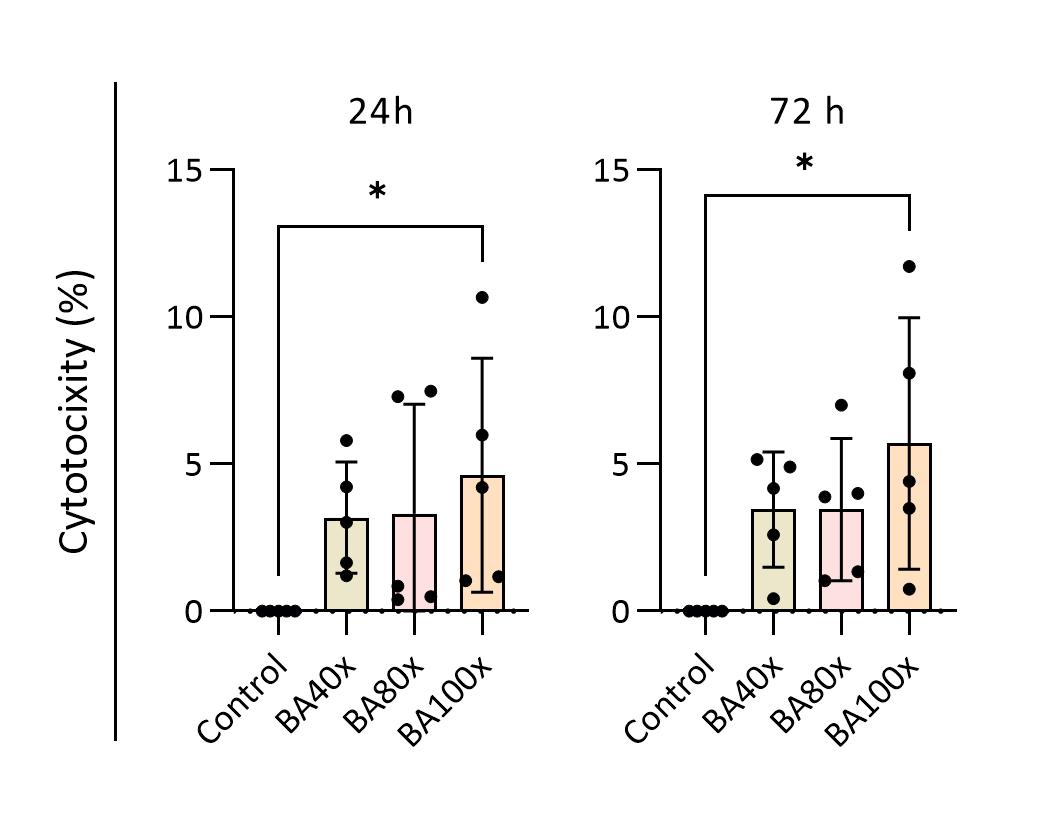
**Supplementary Fig. S1.** Cytotoxicity of BA cocktail in cholangiocyte-like cell organoids (CLCOs).

LDH release as cytotoxicity read-out after exposure of CLCOs to various concentrations of the BA cocktail (for composition see Table 1 of the main manuscript) for 24 and 72 h. Data are presented as mean ± SD of five independent donors. Statistical differences between groups were determined using one-way ANOVA followed by Tukey’s test for multiple comparisons; **P* < 0.05.


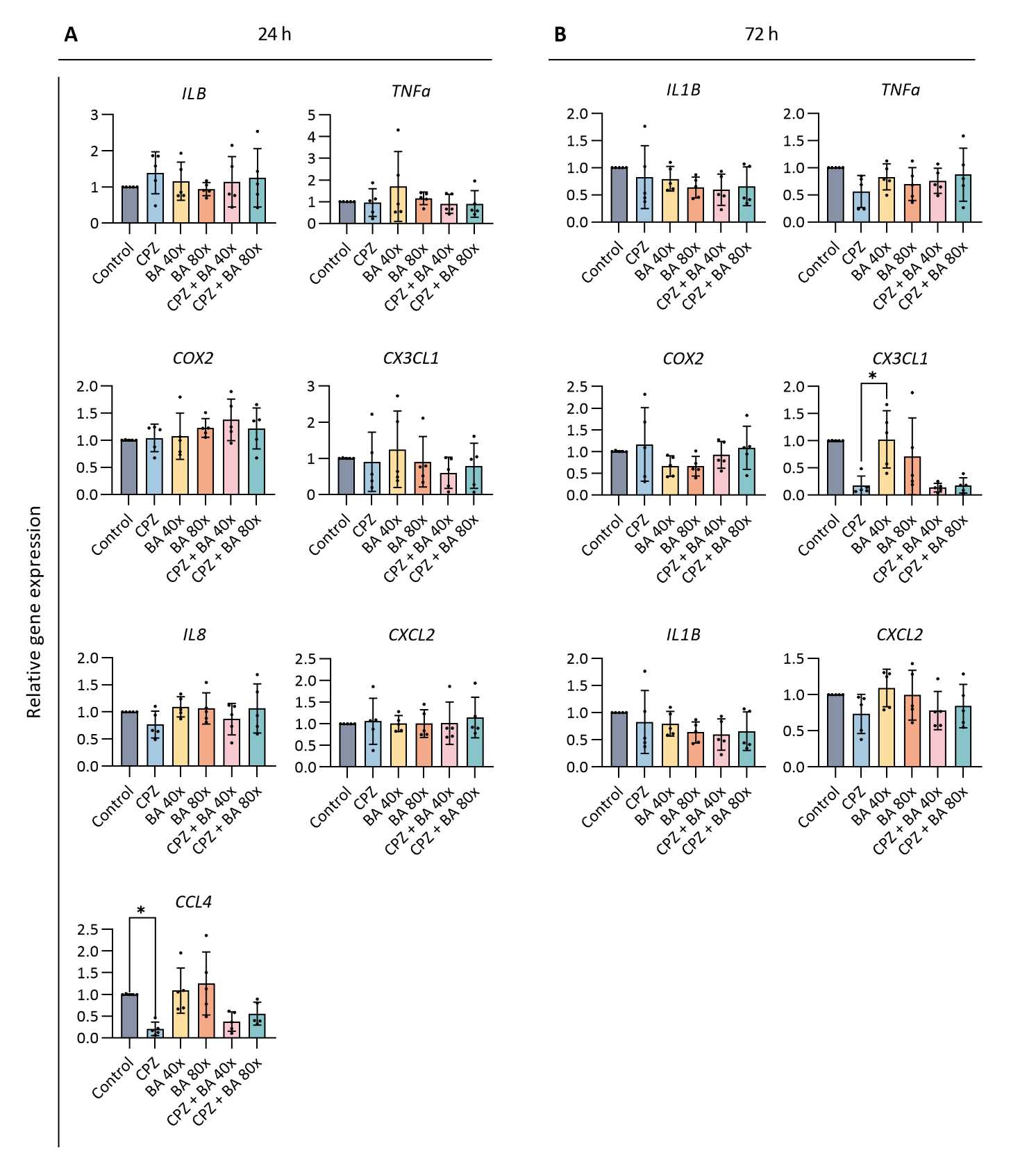


**Supplementary Fig. S2.** Effects of CPZ and BA cocktail on the proinflammatory gene expression.

(A, B) CLCOs were pretreated with solvent (control), 30 μM CPZ with or without BA cocktail (BA 40x or 80x) for 24 (A) and 72 h (B). Gene expressions were estimated by qPCR and normalized to the housekeeping genes. Data are presented as mean ± SD of five independent donors. Statistical differences between groups were using one-way ANOVA followed by Tukey’s test for multiple comparisons; **P* < 0.05.


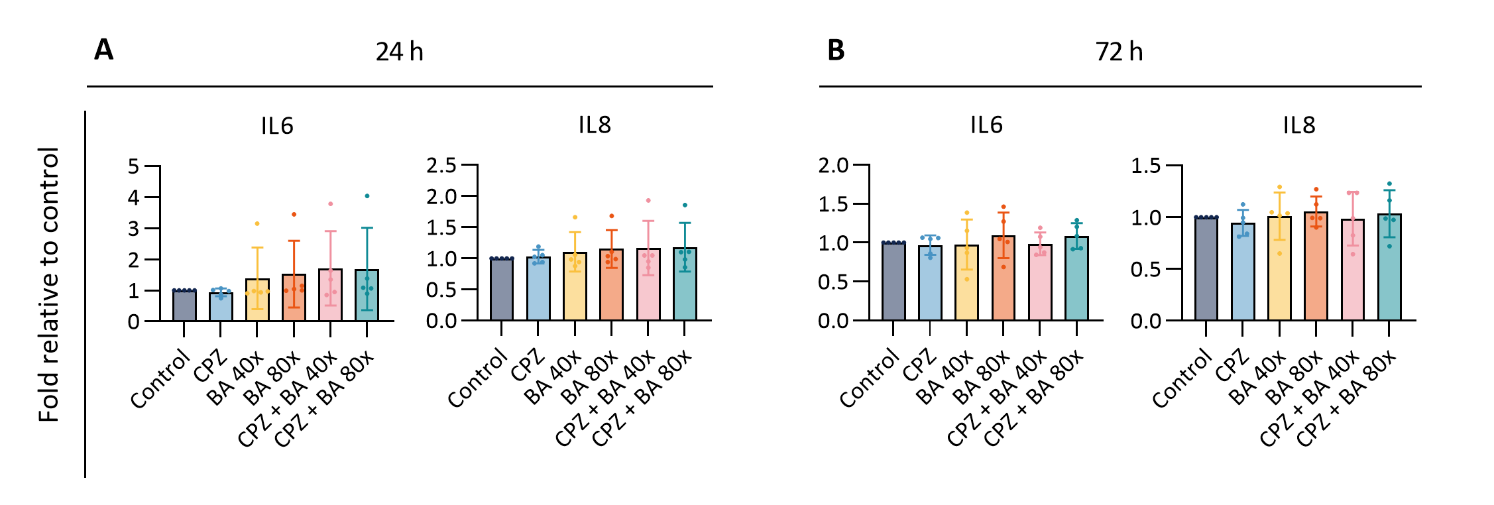


**Supplementary Fig. S3.** Effects of CPZ and BA cocktail on the proinflammatory release.

(A, B) CLCOs were incubated with 30 μM CPZ with or without BA cocktail (BA 40x or 80x) and medium were estimated by ELISA, IL6 and IL8 were tested by 24 (A) and 72 h (B). Data are presented as mean ± SD of five independent donors. Statistical differences between groups were using one-way ANOVA followed by Tukey’s test for multiple comparisons.
